# Health equity principles for oncology real world evidence studies

**DOI:** 10.1093/oncolo/oyae174

**Published:** 2024-07-16

**Authors:** Patrice Forrester, Henry Asante Antwi, Nicholas J Robert, Terri Winston, Amy K O’Sullivan, C Daniel Mullins

**Affiliations:** University of Maryland, School of Pharmacy, Baltimore, MD 21201, United States; University of Maryland, School of Pharmacy, Baltimore, MD 21201, United States; Ontada, Boston, MA 02109, United States; University of Maryland, School of Pharmacy, Baltimore, MD 21201, United States; Ontada, Boston, MA 02109, United States; University of Maryland, School of Pharmacy, Baltimore, MD 21201, United States

**Keywords:** health equity, community health services, health services research, medical oncology

## Abstract

**Background:**

Real-world research on cancer care in the community should address social determinants of health (SDOH) to advance health equity in cancer diagnosis, treatment, and survivorship. We sought patient and stakeholder perspectives to co-develop research principles to guide researchers when using patient record data to address health equity in their research protocols.

**Materials and methods:**

Key informant interviews with 13 individuals elicited perspectives and insights related to health equity and SDOH when conducting research using data from community-based oncology care. Interviews included a brief overview of a prior scoping review and related questions in the interview guide. Key informants included experts in health equity and SDOH, and patient and community advisory board members. Rapid qualitative analysis was used to identify key themes, patterns, and insights from the interview data. Principles were developed based on the results of the analysis.

**Results:**

Three overarching categories for promoting health equity were (1) education; (2) community engagement; and (3) research design and implementation. Education principles highlight the necessity of training in relevant skills to address health equity. Community engagement principles highlight various actions that researchers can take to conduct research inclusive of community concerns regarding health equity. The research design and implementation category provides practical guidelines for researchers in planning, conducting, and disseminating community-based oncology research to address health equity.

**Conclusion:**

Our principles guide oncology real-world research protocols to address SDOH in community settings and promote health equity. These principles should be tailored to specific cancer topics and communities.

## Teaser

Real-world research on cancer care in the community should address social determinants of health to advance health equity in cancer diagnosis, treatment, and survivorship. This study examined patient and stakeholder perspectives to develop principles to guide researchers when using patient record data to address health equity in their research protocols.

**Implications for practice**

There are implications for community oncology research based on study results. Medical providers, particularly those specializing in community oncology research, should be trained in health equity to develop research skills for the promotion of health equity. Community oncology researchers should also advocate for greater patient and community involvement in medical research, including real-world research, to address health equity. Study principles are a living document that can support community oncology researchers in ensuring that health equity is addressed throughout the life cycle of the research process, for a positive impact on marginalized populations in cancer prevention, diagnosis, treatment, and survivorship.

## Introduction

The Centers for Disease Control and Prevention (CDC) defines health equity as “the state in which everyone has a fair and just opportunity to attain their highest level of health.”^1^ Health equity particularly in the community setting is an imperative that demands attention and concerted effort. According to Olanisa et al,^2^ inequities in access to quality cancer care, health outcomes, and the financial costs of treatment remain significant challenges for various marginalized populations (ie, racial and ethnic minorities, individuals living in poverty, rural dwellers, etc.).^3^ Inequities are compounded by the existence of several social determinants of health (SDOH) that hinder access to optimal cancer care. SDOH are the non-medical factors that influence health outcomes. They are the conditions in which people are born, grow, work, live, and age, and the wider set of forces and systems shaping the conditions of daily life.^4^

It has been suggested that real-world evidence holds promise in providing tangible insights for interventions that can improve health outcomes, particularly for groups like African Americans who experience disparities in cancer diagnosis, treatment, and survivorship.^5^ Real-world evidence offers a unique lens through which to gain a deeper understanding of the complex healthcare landscape patients navigate daily.^6^ Derived from routinely collected data, such as claims, billing activities, electronic medical records, and product and disease registries, real-world evidence reflects patients’ daily experiences within the healthcare system. Moreover, real-world evidence helps overcome the limitations of clinical trials because clinical trials may not always include a diverse range of participants, potentially leading to a lack of representation for certain populations, including those from underrepresented ethnic or socioeconomic groups.^7^ On the contrary, real-world evidence draws from a broader and more inclusive pool of patients, reflecting the diversity of individuals who receive healthcare. Thus, real-world evidence can illuminate the root causes of health disparities, identify opportunities for intervention, and guide the development of targeted strategies to narrow gaps in health equity.^2^ These benefits of real-world evidence underscore the growing enthusiasm for research based on real-world evidence, particularly in the realm of cancer care, which demands significant effort to navigate successfully.

While numerous studies have addressed the broader goal of ensuring equity in healthcare in general and cancer care in particular, a growing body of research is actively exploring methodologies to conduct research to achieve health equity, particularly in community.^8,9^ The CDC and the National Academies of Science, Engineering, and Medicine (NASEM) have played pivotal roles in equipping researchers with valuable tools and guidelines to advance health equity research. These tools are designed to empower researchers with community and practice-based strategies, fostering an inclusive and effective approach to public health research. Notable examples include the CDC’s (2013) Practitioner Guide for Advancing Health Equity and the NASEM’s (2017) report highlighting community-based health equity research guidelines. The CDC’s Practitioner Guide for Advancing Health Equity outlines essential skills for public health researchers to incorporate into their work to promote health equity within the public health field. These skills include how to build capacity, engage with communities meaningfully, form partnerships and coalitions, understand health inequities, select research strategies, design and implement plans, and evaluate the research activities.^10^

In this research endeavor, specialists in equity and real-world evidence, alongside patients and community members, aim to co-develop principles that will guide researchers in utilizing real-world data to address health equity in community oncology research. By co-developing principles, we aspire to ensure that research conducted using real-world data is not only scientifically rigorous but also deeply rooted in the lived experiences and needs of the communities it seeks to serve. These principles will serve as a compass, directing researchers toward the path of health equity and illuminating the way forward with a steadfast commitment to achieving equitable health outcomes for all.

## Materials and methods

### Study design and sample selection

This research employs qualitative methods, specifically key informant interviews, to explore perspectives and insights related to how health equity principles can be used by researchers applying real-world data to study oncology care in a community setting. Prior to commencing research, the study was approved and determined exempt by the University of Maryland Baltimore Institutional Review Board (IRB), ID: HP-00104230, ensuring compliance with ethical guidelines and standards for study procedures. A purposive sampling strategy was used to intentionally select participants who could provide valuable insights relevant to the research objective. Participants were recruited through recommendations from either the PATIENTS Program—a community-academic research partnership housed at the University of Maryland Baltimore School of Pharmacy—or Ontada—a company that provides insights and technical solutions for oncology providers. Initial contact was established via email, wherein potential participants were invited by a research team member to take part in the study. The inclusion criteria for the study were patients with lived experience of cancer, or experts on SDOH and health equity. Experts in SDOH and health equity were defined in our study as individuals who have been involved in both cancer research and SDOH or health equity research. In addition, only key informants with demonstrated ability to speak, read, and understand English, and the ability to attend virtual sessions on Zoom were eligible for the study. Conversely, the study excluded individuals who did not meet one or more of the inclusion criteria. This recruitment approach ensured a targeted selection of individuals with diverse perspectives and experiences related to cancer, SDOH, and health equity. Those expressing interest and meeting the inclusion criteria were contacted by a research team member to further explain the research, answer any questions, and schedule the interview sessions. The study strictly adhered to ethical guidelines, ensuring that informed consent was obtained prior to conducting the research. Participants were verbally provided with comprehensive details about the interview process and the content to be discussed at the point of the interview. Prior to commencing the interview, participants were explicitly informed about the nature of the study, and they were asked to provide their voluntary and verbal consent to participate. Additionally, participants were specifically asked for their consent to have the interview recorded, and this consent was also obtained verbally before initiating the interview process.

### Instrument design and data collection

A semi-structured interview guide (see Table 1 for interview guide questions) was designed to explore several key topics, including reactions to findings of a previous scoping review^11^ on SDOH and health equity, new strategies and approaches to address health equity, inequity in electronic health records, and identification of principles needed by researchers utilizing real-world data to address health equity. The interviews were conducted via the Zoom video conferencing platform between March and May 2023. Zoom was also used to record and transcribe interviews. Interviews lasted between 13 and 42 minutes. Participants were at liberty to keep their cameras on or off during interviews. Each interview included an interviewer and a note-taker. The responsibilities of each role were defined to ensure a comprehensive and accurate record of the session. The interviewer conducted the interview by posing interview guide questions and facilitating a comfortable environment for participants to express their thoughts and experiences. The note-taker recorded key points, observations, and non-verbal participant responses during the interview. Throughout the interview process, confidentiality measures were upheld. Participants’ identities and any identifying information were safeguarded to protect their privacy and comply with ethical standards. Data were deidentified after data collection and stored in a password protected file only accessible to members of the research team. Following each interview, the note taker reviewed the interview transcripts generated by Zoom and edited as needed to ensure accuracy of study data.

**Table 1.** Interview guide questions.

Questions
1. I have some questions about your thoughts on the scoping review. What are your reactions to the research findings? 2. The World Health Organization (WHO) is a United Nations agency that promotes health for people around the world. The Centers for Disease Control and Prevention (CDC) is a leading US science-based, data-driven, service organization that protects America from health, safety, and security threats. Health equity is the opportunity for everyone to attain their full potential for health and well-being and to be as healthy as possible according to the WHO and CDC. How do you think health equity could be addressed in community oncology research? 3. When you think about how research has been conducted concerning community oncology, what do you think could be done differently to advance health equity? 4. Electronic health records (EHRs) are sometimes used in community oncology research. Tell me about any examples of racism or other forms of prejudice in electronic health records. 5. What are principles a researcher conducting community oncology research should abide by to address health equity?

### Data analysis

Rapid qualitative analysis was used according to a process described by Hamilton.^12,13^ This analysis aimed to identify key themes, patterns, and insights from the interview data (For a detailed overview of our analysis process, see Table 2). The findings from the rapid qualitative analysis were summarized to provide a concise overview of the key themes and insights that emerged from the interviews. To enhance the rigor and credibility of the study, member checking was performed. On July 6, 2023, participants were sent an email with the summarized results and invited to provide feedback. This member-checking process ensured that the results accurately reflected participants’ perspectives and experiences. Based on the feedback received during the member-checking process, the results were revised as necessary to incorporate participants’ insights and ensure the accuracy of the findings.

**Table 2.** Rapid qualitative analysis process.^[1]^

Steps	Activity
Step 1: Create neutral domain name that aligns with each interview question	One research team member created the following domain names: (1) *Research findings reactions,* (2) *Addressing health equity in community oncology research,* (3) *New ways to advance health equity in community oncology research,* (4) *Electronic health records,* (5) *Community oncology researcher principles to address health equity*
Step 2: Create a summary template	One research team member developed summary template for use by research team. The template included domain names with blank space for summaries of relevant data.
Step 3: Assess summary template for its usability or relevance	The research team (1 postdoc, 1 PhD student) noted relevant data for each associated domain from the first interviewee’s transcript on the summary template. The research team met to discuss the usability and completeness of the summary template domains. The research team came to consensus on the relevance and completeness of the domains.
Step 4: Divide up transcripts amongst research team and summarize data	Twelve remaining interview transcripts were divided amongst research team members. Research team members summarized and noted relevant data on summary templates.
Step 5: Compile summary points into a Matrix	The research team transferred major summary points relevant to understanding of guiding principles needed for addressing health equity in community oncology research into a matrix

^[1]^Information on analysis methods noted in the table can be found in Hamilton (2013, 2020).

## Results

### Participants

Study interviews yielded information regarding researcher principles for addressing health equity in community oncology research using real-world data. Our research team interviewed a total of 13 individuals (see Table 3 for participant information). Study participants included the following: (1) 2 patient advisors who had a prior diagnosis of cancer, (2) 1 community advisor who was the pastor of an African American church, (3) 3 stakeholder advisors inclusive of a clinical university faculty member, a pharmaceutical company representative, and a representative of a medical supplier company, (4) a hematologist oncologist, (5) 1 medical insurance representative, (6) 2 advocates for cancer patients and families, and (7) 3 senior leaders of the same medical supply company that distributes medical supplies, health information technology, and care management tools.

**Table 3.** Study participants.

ID	Role/job position	Type of Organization
1	Hematologist Oncologist	Medical Clinic
2	Patient Advisor	Community-Academic Partnership
3	Senior Scientific Director	Medical Supplier Company
4	Stakeholder Advisor/Faculty Member	Community-Academic Partnership/University
5	Stakeholder Advisor/Senior Manager	Community-Academic Partnership/Medical Supplier Company
6	Patient Advisor	Community-Academic Partnership
7	Vice President	Medical Supplier Company
8	Stakeholder Advisor/Vice President	Community-Academic Partnership/Pharmaceutical Company
9	Insurance Representative	Health Insurance Company
10	Cancer Patient Advocate	Patient & Family Advocacy Group
11	Cancer Patient Advocate	Patient & Family Advocacy Group
12	Community Advisor/Pastor	African American Church
13	Director	Medical Supplier Company

### Researcher principles for RWE studies

Data analysis generated 3 overarching categories for researcher best principles in addressing health equity when utilizing real world data: (1) education; (2) community engagement; and (3) research design and implementation (see Appendix A for summary results). The first category highlights the necessity of training in relevant skills to address health equity. Community engagement principles highlight various actions that community oncology researchers can take to conduct research inclusive of community concerns regarding health equity. The research design and implementation category provides practical guidelines for researchers in planning, conducting, and disseminating community oncology research to address health equity. The following sections detail major themes from the researcher principles document (see Appendix B for details on researcher principles).

### Education

Education principles note that researchers should receive training to obtain competencies in health equity knowledge and cultural sensitivity. This involves researchers gaining the ability to identify and address issues relevant to these competencies. Training in health equity knowledge was particularly important as some study participants with a background in medicine reported that medical researchers often do not understand the concept of health equity and are not trained explicitly in how to address health equity. The vice president of a medical supplier company highlighted this lack of training, saying that medical researchers are “not trained...on health equity... how to do this stuff.” A participant who was a practicing hematologist oncologist affirmed the importance of health equity training: “health equity needs to be incorporated [in] medical education... how to address [health equity] and be aware of it.” The Education category principles also require that researchers gain knowledge of how to access data pertinent for community oncology research addressing health equity.

### Community engagement

Principles in this category focus on eliciting community and patient stakeholder perspectives regarding health equity issues and research plans before, during, and after research. Principles also validate the expertise of patients and key stakeholders on health equity matters impacting them, to be used for the purpose of conducting studies addressing health equity. Patient advisors and the community advisor interviews particularly showed the importance of sharing study results that may have benefit to the population of interest. One of the patient advisors shared that researchers often did not provide the “end result” of a study after getting data from the community.” She emphasized the importance of coming back “to those participants in that community and say[ing] hey, this is what we found. and this is what we recommend, and let’s talk about it.” Related to this, one principle speaks to researchers developing relationships with individuals from populations of interest so as to build partnerships that can sustain community oncology research addressing health equity. This allows the research to have direct benefit for the community, rather than the community “hear about the details [of the research] on the news [after researchers have taken community] information.” (pastor of African American church).

### Research design and implementation

The vast majority of principles are found within the research design and implementation category. These principles reflect the importance of researchers ensuring research plans are patient-centered and address SDOH. For example, one principle states that stakeholder and patient advisors must be a part of the reviewer team when determining whether patient record data should be used by a researcher. An important consideration, particularly for patient and community advisors, was that researchers should examine and address mental and emotional needs of cancer patients through their research. One patient advisor said that “when it comes to health equity it needs to be another key component that needs to be looked at, and that would be mental health...To be treated for cancer...your doctor really needs to also pay attention to your emotional state and to your mental health. and address those needs if necessary.” Principles also note that researchers should abide by national ethical standards such as informed consent and independent review, and have a standardized process for reporting research information. In line with the need to be transparent and ensure that research is truly addressing health equity, it is necessary for researchers to present a publicly accessible final report that gives a summary of their research process and outcomes, including any benefits from the research for addressing health equity.

Patient understanding and accessibility was another large theme in this category. Exemplary principles note the necessity of making sure study results are accessible to populations of interest when possible and are disseminated to organizations that interact with patient populations such as patient advocacy networks. The inclusion of diverse and under-represented groups in studies relevant to the population of the referral geography was another key issue associated with patient access that came up in interviews. It was important to consider diversity and inclusion for patients and for individuals designing and implementing studies. As stated by a patient cancer advocate, “greater efforts to increase involvement in research studies from different groups [representative of the population of interest] would be a continued important initiative... a lot of opportunity for improvements in that area.” Research designers and implementers have a role in assuring patient access through their engagement with patients. Researchers must be respectful of patients and transparent regarding study information requested by patients. In essence, researchers should see their role as not only a designer or conductor of a study but also as one who serves patients through the research process. As one patient advisor emphatically stated, “Researchers need to make us feel valued.... I want to be looked at as a person that has an illness [eg, cancer] that could possibly further research to save people’s lives down the road. Don’t make me feel like you [ie, medical researcher] just recruiting me because I fit your profile. That’s important. Don’t make me feel that way.” Related to this, researchers should not only disseminate study results to physicians, but also to other health care providers such as social workers to inform their engagement of patients for research studies addressing health equity.

## Discussion

We co-developed, with patients and experts in health equity and SDOH, principles to guide researchers in addressing health equity in their real-world evidence research protocols. Principles were based on the perspectives of health equity experts who were patients, providers, community members, advocates, and leaders of healthcare companies. The principles covered the education of community oncology researchers in health equity, engagement of patients and community stakeholders through all phases of research, and practices for ethical and rigorous research that promote health equity. The researcher's principles are important to promoting health equity in community oncology research and can support oncology researchers in gaining access to patient data for their studies. Researchers may use these principles as a guide to address health equity. Research based on these principles may inform interventions that promote better health outcomes for populations (eg, African Americans) impacted by health inequities in cancer diagnosis, treatment, and survivorship.^14^ Findings from this research may also lead to prevention and intervention initiatives that help reduce cancer disparities. In practice, real-world researchers should leverage the SDOH data available to them in medical records, such as race, ethnicity, geographic area, to ascertain any differences in oncology treatment like time to diagnosis or treatment, type of treatment received, and treatment outcomes among diverse patient groups. Once we better understand current differences across groups, interventions may be developed to address health disparities in oncology treatment and bring them to public awareness.

Other researcher principles have been created specific to promoting greater health equity in healthcare as we did, some specific to cancer care. In addition to obtaining the perspectives of key stakeholders and patients, some researcher principles have been formulated through iterative consensus-building activities with community, patient, and researcher networks.^15,16^ Others based principles on previous health equity frameworks or health equity research, exploring how principles could be implemented in research with marginalized populations in contrast to our focus on cultivating the lived experiences of study participants to create principles.^16-18^ Adsul et al, Bailie et al, and Chebli et al’s guiding principles for research addressing health equity in cancer care^15-17^ included similar principles to ours such as the importance of community engagement and prioritization of stakeholder and patient ideas regarding research plans. They also developed principles that did not emerge in our study, related to addressing power relations.^16,17^ In contrast to our principles, there was a strong emphasis on practically addressing health equity issues in the stakeholder communities sustainably, and on explicitly addressing racial inequities in cancer care.^15,17^ The lack of direct emphasis on racial equity and on addressing power dynamics in our principles could be due to the fact that we primarily focused on researcher-initiated studies as opposed to assuming research project plans where researchers and marginalized community members or patients are involved in all aspects of research.

There is literature that similarly notes the importance of community engagement to health equity researcher principles as we did. Emmons et al highlighted the importance of community engagement to address health equity in the process of cancer research.^19^ Further, he suggests data sharing or dissemination, as was suggested by participants in our study, to ensure both community and researcher understanding of the issues involved and move toward shared benefits. Instead of formulating principles, though, Emmons et al evaluated and critiqued community engagement researcher principles developed by NIH with a view to strengthening greater equity in the data-sharing process.^19^ Similarly, one of the patient advisors who is the author of this paper, related that our researcher's principle regarding the submission of a final research report should not only be disseminated in ways that providers or researchers understand but should also be disseminated in ways that are understandable to the communities that are the focus of the study. Kruse et al show how community engagement principles as created by the Clinical and Translational Science Awards Consortium’s Community Engagement Key Function Committee Task Force on the Principles of Community Engagement (2011) can be implemented in community cancer centers in a particular locality—such as the city of Boston.^20^ These principles focus explicitly on community engagement practices for research as we did but provide greater detail about understanding the context of the populations of interest to promote long-term health change. As we noted in our principles, it is very important to engage patients when conducting community oncology research which was also affirmed in Charlot et al^21^ Charlot et al found that patient advisors for research projects focusing on cancer can support patient enrollment in studies, including clinical trials.^21^ There were also some challenges to engaging patients, although, that we did not consider in our principles which should be assessed when implementing principles. Charlot et al had difficulties with retaining patient advisors due to some of them having to attend to their own health treatment and due to a lack of balance between English and non-English speakers on the advisory board.^21^ A balance of patient advisors regarding relevant social identities and developing creative ways to involve patients in advisory boards that take account of their healthcare needs may need to be considered when recruiting and engaging patient advisors.

Education of community oncology researchers was an important insight noted by participants in this study. However, more literature focuses on educating providers in the medical field than medical researchers in terms of addressing health inequities and communicating with patients about inequities.^22^ Relevant to education is the importance of researchers gaining skills in cultural sensitivity. In line with our principles regarding training in cultural sensitivity, Lett et al notes the importance of self-reflection on biases as a safeguard for perpetuating further inequities in healthcare research.^18^ In conducting research, we also note the importance of attending to diversity and inclusion for patients and research staff in researcher principles to address health equity. Although not specific to cancer care, researcher commentary speaks to the importance of diversity and inclusion in research addressing health equity. Fleming and Burgette note that diversity and inclusion in research teams lead to more innovative and relevant science that connects with populations of interest.^23^

### Strengths and limitations

Our research has the distinction of not using any pre-determined framework to develop principles, but rather we examined the lived experiences of community oncology stakeholders regarding the necessary guidelines for addressing health equity in oncology research. Particularly noteworthy were the insights gained from medical practitioners and researchers who shared that lack of training in health equity for medical providers could make it difficult for them to even know how to address health equity through their research. As such, these principles may be highly important as guidelines for medical providers engaging in this type of research. Limitations of this research are that we did not include a large or balanced amount of community and patient advisors from various social groups, which may have provided a greater understanding of health equity principles needed for different communities. For example, a patient advisor on our study reported that future research principles should be explored concerning full disclosure to community members of who comprises the research team and self-disclosure of researcher experiences (eg, caregiving for family member with cancer) within boundaries that may connect with patients and others when engaging communities. In addition, the patient advisor stated that a plan needs to be developed for how to implement the principles regarding health equity education. It is also important to evaluate the usefulness of these principles for community oncology research in other contexts, such as clinics, hospitals, and other organizations who interface with cancer patients but have little to no cancer research expertise.

## Conclusion

We developed guiding principles for researchers to address SDOH in real-world oncology research protocols in order to promote health equity. Our principles require researchers to obtain proper education to address health equity in research, engage communities and patients as part of the research process, and design and implement research studies that address SDOH and promote health equity. Researcher principles should be operationalized and evaluated, to ensure they align with the goal of health equity promotion in community oncology research. Researchers utilizing our principles to promote health equity should tailor their research approach to the specific research context, drawing upon the expertise of relevant patients and key stakeholders. The researcher principles can also be used as a template for others who may want to co-develop guiding principles for researchers to promote health equity in other areas of health research. As we did in this study, patients, community members, and other experts in health equity and SDOH can provide their perspectives on necessary researcher principles to promote health equity, using our researcher principles as a basis for discussions.

## Data Availability

The data underlying this article will be shared on reasonable request by the corresponding author.

## Appendix A

### Rapid qualitative matrix

**Table:**

	Research findings reactions	Addressing health equity in community oncology research	New ways to advance health equity in community oncology research	Electronic health records	Community oncology researcher principles to address health equity
** ID**					
1	Consistent with research participant conducted There are also trust issues	Importance of looking at subgroups in cancer research Data driven “strong policy initiatives” to develop this research	Education in medical training about how to address health equity Need to be incorporated into medical professional society treatment guidelines	Limited data sets because physicians often blinded to patients’ social situation Reach out to communities themselves to get a fuller picture; the patients in the record may not represent full community Comprehensive analysis of billing data, looking at zip code distribution where patients being referred from	Structure studies to look at health equity “Provide translation services, have our clinical protocol and the consent form in the patient’s native language.” Make sure representation of patients in study is similar to makeup of referral geography Be respectful, be aware of some of the ethnic practices of people
2	“The results are not surprising. Both the system and the individual’s reluctance to seek healthcare and their lack of trust in healthcare providers are responsible for this problem kind of results”	Partnership with community or opinion leaders (eg, churches, community health workers, etc.) since community members are more comfortable with them Having time to listen to community members and mentoring them to become health conscious. Allows the patient to actually have their input in the health research process.	Let people participate in research wherever they are including marketplace, parole and probation office, Barbershops, Beauty salons, grocery stores etc. Follow up on research to let participants in that community know what you found and your recommendation.	“America is so caught up on race and race makes a difference in everything we do. So even electronic records.” If EHR does not ask about race, zip code etc. it will reduce prejudice and stigmatization	Researchers must respect everybody regardless of race, socioeconomic status etc. “Before the actual research takes place come into the community and talk to people, get their trust...Use town hall meetings, little block party appreciation, going to the local school and participating in something, getting books for the library, or having some of your staff come in and read to the children.”
3	“we did a study on systematic literature review of uses of social determinants of health. In early stage cancers… we had similar...” “I’m a little surprised that you only have 2 variables under the compliance where you have 5 under the access to care - I’m thinking why would somebody be non compliant? Well, then they can’t go back.”	“NCCN -stretch thermometer distress... what it measures is… individual person level and not just social determinants of health, but psychological determinants of health.” Retrospective research on treatment—is there differentiation in level of tx prescribed?	More research that explicitly must include an exploration of SDOH impacts	Limited in SDOH get from EHR—mainly get race and payer status	Researchers should be aware of the ecological fallacy—attributing characteristics of a group to an individual. Always include SDOH variables in study
4	“Not surprised but I thought that transportation and food will be the top SDOH”	The work required to address SDOH needs a lot of time and resources Integration of social workers within the practice	Invest in building clinical research infrastructure in the community Stimulate growing awareness that SDOH is important	Finding ways to allow researchers to flag certain words that constitutes prejudice	Researchers must first develop the skill set for both community engagement and understanding what equity is Know how to find information within the EMR. Every EMR has information structured in a different way. So you’d want to know how to access the data on the front end
5	“Not surprised by the results. I think people are now getting to tackle some of the deeper problems that we have always talked about from the list of SDOHs that are studied these days”	We have so much data already from past studies but we are not doing anything with it. We should do something with it	We need to go to the communities and let them know the importance of the research and seek their opinion on the design and implementation of the research	I don’t know much about the specific issues with the EHR but I appreciate any study that will bring these issues out.	The researchers need to develop skills to better engage communities, especially communication skills to motivate participation in research
6	Need to also examine mental health needs of cancer patients	Understanding mental and emotional needs of patients in addition to physical needs Understand differences within ethnic groups Explore how doctors can communicate with their patients so that patients feel heard; how does that impact access to care? “I think researchers need to find out what patients feel, how they felt, what they wish they had.. what they wish they didn’t have.”	Think of ways to involve people of color more in the research, clinical trials Researchers should be transparent and make patients feel valued “I want to be looked at as a person that has an illness that could possibly further research to save people’s lives down the road. Don’t make me feel like you just recruiting me because I fit your profile. That’s important. Don’t make me feel that way.”	No experience of racism in EHR	Need to remember that the goal of the research is to address health equity Write a summary report: “I think they should write some type of report, and it doesn’t have to be a long report. What were the benefits of them using that data, what would they have liked to see more? What would they like to have seen in the future to help them with their research.” Don’t have person’s name attached to data record Have patients be a part of the process of choosing whether researchers can use data.
7	Not surprised by research findings	Hard to address because don’t have ability to address aspects of health equity such as food insecurity as researcher “We can’t fix other…societal drivers of healthcare. It’s just not in our control, nor do we have funding to be able to do that. So I think we can…try our best. But we have to rely on other resources to be able to solve this problem here.”	Understand better what patient needs are Document and talk about SDOH to better understand the problems Think about how to address health equity as a society differently; usually society focuses on band-aid solutions like charities, etc.	EMR only includes 2 sexes—male and female; does not cover other genders Participant’s organization is releasing some upgrades in EMR that include preferred pronouns, gender identity	Need for training in how to address health equity in research Make sure following written ethical guidelines that abide by national standards for research
8	Not very surprised with results but thought the duration of the recurrence of cancer and its relationship with SDOH is interesting. Can we show that income and social protection, or access to affordable health service of decent quality is clearly linked to disease recurrence? That will also be interesting.	Ensuring that underrepresented populations are included in the research	We need to make sure that both [patients] who come to the clinic and those who are unable to come to the clinic because of other commitments are included There’s a mistrust of research in a lot of the population. So that narrative needs to be fixed	There is talk of Artificial Intelligence (AI). Some institutions are using AI to help pick up on some of these terms that are written within the text Trying to promote prompts or tools, or whatever within the actual EHR that captures the data	More systematic and guided way of capturing research information and design of the History of Present Illness (HPI) templates to make it more equitable
9	Structural conflict as a SDOH was interesting because had never thought of that Thought other findings were accurate	Utilizing social workers to help with minimizing barriers to getting into community oncology care/research Medical providers/researchers need to know better how to address patient barriers	Needs to be more diverse representation of income levels, geography, and housing statuses in research Think about intersectionality in research—different identities that people hold at the same time Give participants with barriers to enrollment a certain amount of money to overcome those barriers (eg, money for transportation)	Errors in data entry for EHR Issues around identity -sometimes people who are ethnically Hispanic are assumed to speak Spanish when they do not Don’t have categories for sexual orientation in EHR Look at in-group differences	“How can they (researcher) overcome a barrier they don’t understand?” Become aware of health equity issues and self-aware Go through training to understand reasons for patients not enrolling and how to address
10	Not surprised by findings Ranking SDOH instead of just identifying them was interesting	More needs to be done to include analysis of access in different populations Diversity planning in research protocols and diversity strategies from early on in the pre-planning stage	Additional care facilities to support research infrastructure Personnel trained and working with patients and families about participating in clinical research Support staff such as additional study managers to review and implement clinical studies, data managers to help with data collection, electronic infrastructure to collect data in ways required by clinical trials	Have different patient groups review language in EHR to identify and remedy bias	External review Make sure people involved in designing and conducting clinical trials are representative of community being studied Increase involvement in studies from diverse groups Review consent documents so that they are usable and understandable for study participants Collect patient characteristics in an accurate and standardized manner; increase rigor and accuracy of data collection
11	“I am surprised why housing and basic amenities does not impact adherence”	We have to look at variations of SDOH by geography and account for them in the design of the study. For example in some places it might be literacy. In some places, it might be food insecurity	People are conflicting diversity with health equity and they’re really different things with different problems and we need to make sure we understand it very well so that we do not mis- design the research.	The original purpose of EHR is for billing not research hence the information collected is suitable for billing. There are bound to be challenges when it has been used for other purposes and that will be difficult to completely eliminate.	There is a need to create a manual to guide the use of this research. The manual should provide indicators to flag studies that are clearly not focused on equity
12	Not surprised at results	Include minority populations earlier in the research process	Include more Black people and other minorities in studies Diminish barriers to being involved in research for minority communities by involving trusted messengers in the process earlier on (eg, pastor, community leader) who can share information on the research process	Need to understand the purpose of using “offensive” language in EHR Be transparent about how these “offensive” terms used in EHR impact certain communities disproportionately Focus on how to address issues that disproportionately affect certain communities, including pre-disposing factors that lead to cancer	Be mindful role is also serving study participants View research through an equity lens from beginning Be a person that participants trust—cultivate relationships with trusted messengers in the community and participants Ensure results shared with community so that it can have benefit on the community studying in research Training on how researcher addresses challenges that impact cancer patients such as mental health issues, training on cultural sensitivity, training on how to address myths around cancer/cancer research
13	I understand all of them. Not surprised	We need to better understand social drivers of health and what is needed to mitigate those	We need to expand the parameters of a study to capture more diverse people Researchers must go through some training about what equity itself is and not just assume that anybody understands the concept of equity.	I do not have a specific comment but I have heard there are issues with them but I do not have any evidence of it	Having principles to check if a study is focused on equity will not be necessary because it will limit innovative thoughts and research activities. However, if I can give some suggestions, as follows. We need to keep creating awareness of the problems of inequity Researchers must prove that they have received training in equity before getting access to the data There should be a way to vet their studies by filling forms that measure the equity index of the study

## Appendix B

### Health equity principles for real world evidence studies

#### Education

##### Training in health equity

Researchers must show evidence that they have received training in health equity.

Researchers should acquire the following competencies:

##### Health equity knowledge

Researchers must be able to define health equity.

Researchers must have a working understanding of how to address health equity.

##### Patient enrollment

Researchers must be able to identify barriers to patient enrollment in studies addressing health equity.

Researchers must show an understanding of how to implement interventions to address barriers to patient enrollment in studies addressing health equity.

##### Cultural sensitivity

Researchers must be able to define cultural sensitivity—the knowledge, skills, attitudes, and beliefs that enable community oncology researchers to effectively engage and respond to diverse patients, caregivers, and providers as they conduct studies (American Academy of Family Physicians, 2008).

Researchers commit to identifying how diversity in cultural values and context of the population(s) of interest in the study could impact study results.

Researchers commit to self-reflection on how implicit biases or stereotypes could impact study protocols or interpretation of results.

#### Data access

Researchers must demonstrate understanding of how to access information in the patient data record for the use of conducting future studies addressing health equity.

### Community engagement

#### Pre-engagement

Whenever possible, researchers should commit to participating in community events that benefit the health of patient and stakeholder communities. This will help build future partnerships.

#### Patient and stakeholder involvement

Researchers commit to involving patients as partners rather than as “human subjects.”

In research projects, researchers commit to involving patients, trusted messengers (eg, community leaders, religious leaders, family members), and other stakeholders as advisors.

#### Developing research with input from patients and stakeholders

Researchers must identify the health equity concerns of patients and stakeholders before planning studies addressing health equity.

Researchers should explore patient and patient advocate ideas regarding implementation of research addressing health equity.

#### Research dissemination

Researchers should share study results with relevant patients and stakeholders so that it may benefit populations examined in the study.

Researchers commit to providing recommendations based on study results to communities that may benefit from study findings. Results and possible benefits of results to the community should be reviewed by patients and patient advocates to ensure the language used is meaningful.

### Research design and implementation

#### Patient-centered research addressing social determinants of health)

Researchers must demonstrate that research design and implementation are patient-centered and address social determinants of health (SDOH).

#### External review

Proposed studies must be reviewed externally by patient reviewers or a stakeholder advisory board to ensure they are patient-centered and address SDOH.

Study research protocols and consent documents should be usable and understandable for study participants (eg, provide translation).

Reviewers vet studies by filling out forms that measure health equity index.

#### Research support staff

Whenever possible, researchers should integrate social workers or other support staff into their studies to help address health equity issues impacting patients and minimize patient barriers to research participation.

#### Study location

Whenever possible, researchers should develop research designs that allow for studies to be conducted within study participants’ community (eg, barbershops, grocery stores, religious organizations, and beauty salons).

#### Diversity and inclusion

Study participants must be similar to the population of referral geography.

Researchers must maximize study inclusion criteria, increasing the involvement of diverse and under-represented groups (eg, different income levels, housing statuses, races/ethnicities).

Whenever possible, the designers and implementers of the study should be representative of the population(s) of interest in the study.

#### Patient engagement

Study designers and implementers must be respectful of patients.

Study designers and implementers must be transparent regarding information relevant to the patient when patients request information concerning their participation.

Study designers and implementers should view patients as having dignity and worth.

Study designers and implementers should view their role as serving patients through research activities.

#### Data collection and use

Researchers should not attach study participant’s personal identifiable information to data records.

Researchers must follow written ethical guidelines that abide by national standards for research.

Researchers must collect research information in an accurate and standardized manner.

Whenever possible, researchers should examine subgroups or within-group differences as relevant to their study.

Whenever possible and relevant, researchers should examine and address the mental and emotional needs of cancer patients in addition to physical needs.

Researchers should be aware of the ecological fallacy—attributing characteristics of a group to an individual.

#### Research report

Researchers must submit a study report to Ontada that gives an overview of the research process and outcomes, including any benefits from use of Ontada’s data for promotion of health equity.
